# Asthma Powders

**Published:** 1907-06-15

**Authors:** 


### Points in Treatment.

### ASTHMA POWDERS.

Asthma is a loose term amongst the laity, used to describe almost any attack of wheeziness or cough, particularly if it be intermittent. True asthma is not extremely common, though many conditions are diagnosed as such. There is no doubt, however, of the efficacy of certain fumes in the relief of a paroxysm of true asthma, and not a few of the other paroxysmal affections of breathing may be mitigated in the same way. This fact has been known for ages, and many a country woman can make up the powder which she burns under her husband's nose to relieve his asthma. Such powders have been patented under various names, and some of them have undoubted efficacy; but every medical man should be able to prescribe his own asthma powders, and, if necessary, dispense them, varying the ingredients and their proportions according to the needs of his individual patients.

Most hospital pharmacopœias contain formulæ for asthma powders, but few men can write the prescription for one from memory. This is a pity, since it leads to the use of patent powders whose composition may be uncertain in the doctor's mind.

Almost all asthma powders contain potassium nitrate, the purpose of which is simply to ensure a continuous lively smouldering of the lighted mixture, with evolution of abundant fumes; most of the powders contain either lobelia or stramonium, or both; these are the source of the active anti-spasmodic principle given off in the fumes. The powders also contain some other vegetable basis whose nature does not matter much so long as the smoke it gives off is not too pungent or unpleasant. Sometimes black tea is used, but more often powdered aniseed.

A well-known prescription is the following:—

℞ Potassii nitratis ... ... ... ... ʒj.

Pulveris Lobeliæ inflatæ ... ... ... ʒj.

Pulveris stramonii foliarum ... ... ʒj.

Pulveris anisi fructûs ... ... ... ʒj.

Solve potassii nitratem in aqua ℥j.; adde pulveres lobeliæ, stromonii et anisi; misce; evapora lente; fiat pulvis.

It is not so good merely to mix the dry powders together as they are as to mix them into a paste in a solution of the potassium nitrate as above directed, and then obtain the powder by allowing the paste to dry; the mixture is much more intimate, and the burning of it is much more equable and free from startling fizzes and spurts when the dry process is employed. If the powder is needed urgently, however, and none is to hand ready prepared, simple mixing of the dry ingredients is permissible.

In using the powder about a teaspoonful of it is measured out on to a plate or saucer, in a room where there is no draught to blow the powder about. It should be pinched together into a conical pile and a match applied to the apex of the cone. The powder will burn steadily and moderately rapidly, giving off not unpleasant fumes. It is these fumes that the patient inhales as the plate with the burning powder is held about a foot or less beneath his nose.

The fumes of the powder relieve the immediate distress in many cases, but their effect is apt to be transient. The other drug which seems to do most good in relieving the spasmodic contraction of the bronchioles in true asthma is belladonna; its effect comes on much more slowly than does that of the fumes, but it lasts a great deal longer. At the same time as giving the fuming inhalation, therefore, it is well to give a hypodermic injection of liquor atropinæ sulphatis. Different people are so very different in their reactions to belladonna or atropine that, in a patient in whom the effects are not already known, it is best to begin with as little as $\frac{1}{300}$ grain of atropine sulphate; but in many cases it is not until $\frac{1}{100}$ grain, or even $\frac{1}{5}$ grain, has been given that the expected benefit will be obtained.

